# l-Citrulline Metabolism in Mice Augments CD4^+^ T Cell Proliferation and Cytokine Production *In Vitro*, and Accumulation in the Mycobacteria-Infected Lung

**DOI:** 10.3389/fimmu.2017.01561

**Published:** 2017-11-16

**Authors:** Shannon M. Lange, Melanie C. McKell, Stephanie M. Schmidt, Austin P. Hossfeld, Vandana Chaturvedi, Jeremy M. Kinder, Jaclyn W. McAlees, Ian P. Lewkowich, Sing Sing Way, Joanne Turner, Joseph E. Qualls

**Affiliations:** ^1^Laboratory of Dr. Joseph E. Qualls, Division of Infectious Diseases, Department of Pediatrics, Cincinnati Children’s Hospital Medical Center, Cincinnati, OH, United States; ^2^Immunology Graduate Program, University of Cincinnati/Cincinnati Children’s Hospital Medical Center, Cincinnati, OH, United States; ^3^Laboratory of Dr. Joanne Turner, Department of Microbial Infection and Immunity, College of Medicine, The Ohio State University, Columbus, OH, United States; ^4^Laboratory of Dr. Sing Sing Way, Division of Infectious Diseases, Department of Pediatrics, Cincinnati Children’s Hospital Medical Center, Cincinnati, OH, United States; ^5^Laboratory of Dr. Ian P. Lewkowich, Division of Immunobiology, Department of Pediatrics, Cincinnati Children’s Hospital Medical Center, Cincinnati, OH, United States; ^6^Texas Biomedical Research Institute, San Antonio, TX, United States

**Keywords:** l-citrulline, l-arginine, T cells, argininosuccinate synthase, argininosuccinate lyase, arginase, mycobacterium, tuberculosis

## Abstract

Activation, recruitment, and effector function of T lymphocytes are essential for control of mycobacterial infection. These processes are tightly regulated in T cells by the availability of l-arginine within the microenvironment. In turn, mycobacterial infection dampens T cell responsiveness through arginase induction in myeloid cells, promoting sequestration of l-arginine within the local milieu. Here, we show T cells can replenish intracellular l-arginine through metabolism of l-citrulline to mediate inflammatory function, allowing anti-mycobacterial T cells to overcome arginase-mediated suppression. Furthermore, T cell l-citrulline metabolism is necessary for accumulation of CD4^+^ T cells at the site of infection, suggesting this metabolic pathway is involved during anti-mycobacterial T cell immunity *in vivo*. Together, these findings establish a contribution for l-arginine synthesis by T cells during mycobacterial infection, and implicate l-citrulline as a potential immuno-nutrient to modulate host immunity.

## Introduction

*Mycobacterium tuberculosis* (*Mtb*) infection continues to plague global health, yet treatment and prevention methods targeting tuberculosis (TB) disease has generally remained stagnate in the last several decades. Although current antibiotic regimens have revolutionized TB control, patients burdened with multidrug-resistant strains of *Mtb* are left with few treatment options (1). Host-directed therapeutic strategies offer a novel concept to boost protective immunity in conjunction with targeting the pathogen itself, warranting investigation into regulatory mechanisms of key immune responders to mycobacterial disease.

CD4^+^ T cells are essential for anti-mycobacterial immunity, evident by the drastic increase in disease susceptibility in patients co-infected with *Mtb* and HIV (2, 3), and in transgenic mice lacking components of the CD4^+^ T cell response (4–8). Upon activation in the lung draining mediastinal lymph nodes (mLNs), proliferation and migration of T cells to the lung facilitate activation of infected macrophages *via* secretion of inflammatory cytokines, such as IFN-γ. Intriguingly, this vital response centers on the availability of the amino acid l-arginine (9). When l-arginine is limiting in the microenvironment, T cells become hyporesponsive to stimuli—ceasing proliferation (10–13), cell cycle progression (14, 15), and cytokine production (12, 16). Myeloid cells actively inhibit T cells in this fashion by expressing the urea cycle enzyme arginase 1 (Arg1) to locally deplete l-arginine (17–19). During mycobacterial infection in mice, Arg1 activity suppresses T cell activity (20, 21) and correlates with decreased T cell responsiveness in TB patients (20), creating a metabolic hurdle for protective T cell immunity.

Despite this suppressive mechanism, T cells have acquired the ability to synthesize intrinsic l-arginine from the ubiquitous, non-canonical amino acid l-citrulline through the sequential activities of argininosuccinate synthase (Ass1) and argininosuccinate lyase (Asl) (22). We have previously demonstrated the necessity of l-citrulline metabolism for host defenses against mycobacterial species in macrophages *in vitro*, and in the broader hematopoietic population *in vivo* (23, 24). T cells also harness l-citrulline for proliferation and reversal of hyporesponsiveness (11, 13, 14, 25), yet little is known on how this metabolic pathway impacts T cell activity driven by mycobacterial infection. In this study, we uncover the contribution of l-citrulline metabolism on CD4^+^ T cell functions in the context of mycobacterial infection. Our data reveal T cells rely on l-citrulline in microenvironments limited in l-arginine to maintain proliferation and cytokine production. Finally, these *in vitro* observations led to the discovery that l-citrulline metabolism is required for local CD4^+^ T cell accumulation during mycobacterial infection *in vivo*.

## Materials and Methods

### Mice

Mice were bred within the Division of Veterinary Services at Cincinnati Children’s Hospital Medical Center (CCHMC). Strains are available from The Jackson Laboratories [C57BL/6J, 000664; B6.129S7-*Asl^tm1Brle^*/J, 018830; B6.Cg-Tg(Tek-cre)1Ywa/J, 008863; C57BL/6-Tg(H2-Kb-Tcra,-Tcrb)P25Ktk/J, 011005; C57BL/6-*Arg1^tm1Pmu^*/J, 008817 (originally a gift from Peter Murray, St. Jude Children’s Research Hospital); B6.Cg-Tg(Cd4-cre)1Cwi/BfluJ, 022071] and Charles River Laboratories (B6.SJL-*Ptprc^a^Pepc^b^*/BoyCrCrl, 564).

### Tissue Culture

Complete RPMI 1640 (C-RPMI, 1.14 mM l-arginine, no l-citrulline, 10-040-CV, Corning Cellgro) was prepared by addition of 10% fetal bovine serum (SH30071, GE Healthcare) and 1% penicillin/streptomycin (15140-122, Gibco, Life Technologies). l-arginine-free C-RPMI 1640 (R-free RPMI, 89984, Thermo Scientific) was supplemented with 10% dialyzed fetal bovine serum (35-071-CV, Cellgro, Corning Life Sciences), 1% penicillin/streptomycin (15140-122, Gibco, Life Technologies), and l-lysine (L8662, Sigma-Aldrich) to match the formulation of C-RPMI. R-free C-RPMI was supplemented with l-arginine (A8094, Sigma-Aldrich) or l-citrulline (C7629, Sigma-Aldrich) prepared in sterile water (100 mM stock) at 1 mM final concentrations.

### Mycobacterial Infection and Colony Forming Unit (CFU) Enumeration

*Mycobacterium bovis* BCG infection: *M. bovis* bacillus Calmette–Guérin Pasteur strain was cultured in Middlebrook 7H9 broth (M0178, Sigma-Aldrich) supplemented with 0.05% tween-80 (P4780, Sigma-Aldrich) plus OADC enrichment (R450605, Thermo Fisher Scientific) at 37°C shaking ~50 r.p.m. Bacilli were washed twice with sterile PBS prior to use. For *in vitro* studies, bacilli were heat-inactivated (HK-BCG) by incubating at 65°C for 30 min and plated on Middlebrook 7H10 agar (262710, Difco) supplemented with OADC enrichment for 3 weeks at 37°C to confirm sterilization. For *in vivo* infection, anesthetized mice were inoculated with approximately 5 × 10^6^ bacilli by intranasal administration. At 8 weeks postinfection, tissues were harvested and processed for analysis. Infected lung tissue was homogenized in 5 ml sterile PBS and serially diluted on 7H10 agar supplemented with 2.5 mg/l amphotericin B (A9528, Sigma-Aldrich), 200,000 U/l polymyxin B sulfate (P4932, Sigma-Aldrich), 20 mg/l trimethoprim lactate (T0667, Sigma-Aldrich), 50 mg/l carbenicillin (C3416, Sigma-Aldrich), and OADC enrichment. CFUs were quantified following 3 weeks at 37°C. To harvest live mammalian cells, lungs were digested for 1 h at 37°C in DMEM (10-013-CV, Cellgro, Corning Life Sciences) supplemented with 10% bovine calf serum (SH30073.03, Thermo Fisher Scientific), 1% penicillin/streptomycin (15140-122, Gibco, Life Technologies), 0.5 mg/ml deoxyribonuclease I (LS002139, Worthington Biochemical Corporation), and 1 mg/ml collagenase (C7657, Sigma-Aldrich). Lung digests and mLNs were processed into single cell suspensions and stained for flow cytometry. *Mtb* infection: *Mtb* Erdman (35801, American Type Culture Collection) was grown in Proskauer–Beck liquid medium containing 0.05% tween-80 to mid-log phase and frozen in 1 ml aliquots at −80°C. Mice were infected with *Mtb* using an inhalation exposure system (Glas-col) calibrated to deliver 50–100 CFU to the lungs of each mouse, as previously described (26). At day 30 postinfection, mice were sacrificed and lungs were aseptically removed into sterile saline and homogenized. Serial dilutions were plated on 7H11 agar supplemented with OADC. Plates were incubated at 37°C for 3 weeks to enumerate bacterial colonies and calculate bacterial burden.

### Macrophage Preparation

Mice were injected i.p. with 1 ml sterile thioglycollate (R064710, Thermo Fischer Scientific). Peritoneal exudate cells were collected after 4 days by lavage, followed by red blood cell lysis and plating on 96-well round bottom plates at 1.4 × 10^5^ cells/well. Following adherence, macrophages were stimulated with HK-BCG representing an MOI = 20 to yield consistent T cell stimulation. In some experiments, arginase activity was induced by overnight prestimulation with 10 ng/ml each mouse recombinant IL-4 and IL-10 (14-8041-62 and 14-8101-62, eBioscience). The following day, C-RPMI containing non-adherent cells was aspirated, cells were washed with PBS to remove remaining l-arginine-containing medium, and R-free C-RPMI was added.

### T Cell Proliferation Assay

Peripheral lymph nodes and spleens were harvested from naïve mice and processed to a single cell suspension. Following red blood cell lysis, lymphocytes were incubated with 2 µM carboxyfluorescein succinimidyl ester (CFSE, Sigma-Aldrich #21888) for 10 min followed by addition of 20% fetal bovine serum v/v in PBS. T cells were plated at 2 × 10^5^ cells/well and stimulated by α-CD3 (BE0002, BioXCell) and α-CD28 antibodies (BE0015.1, BioXCell), p25 peptide (FQDAYNAAGGHNAVF, UBR140905A-1, United Biochemical Research), or HK-BCG-stimulated macrophages in the indicated l-arginine or l-citrulline conditions. In some experiments, cells were supplemented with 2-mercaptoethanol at 55 µM final concentration to promote cell viability. HK-BCG-stimulated wells contained the nitric oxide synthesis inhibitor *N*-[[3-(aminomethyl)phenyl]methyl]-ethanimidamide, dihydrochloride (1,400 W, 100 µM final concentration, 214358-33-5, Cayman Chemical) to preserve T cell viability, and indicated wells were supplemented with S-(2-boronoethyl)-l-cysteine (BEC, 250 µM final concentration, 63107-40-4, Cayman Chemical) to inhibit arginase activity. T cell proliferation was analyzed by flow cytometry after 72 h in culture. Proliferation index represents the inverse of the mean fluorescent intensity of CFSE within CD4^+^ T cells normalized to the l-arginine/l-citrulline free culture condition.

### T Cell Polarization

Single cell suspensions of lymphocytes were obtained as above. Lymphocytes were plated at 2 × 10^5^ cells/well in R-free RPMI with 1 µg/ml α-CD3 supplemented with 1 mM l-arginine, 1 mM l-citrulline, or neither amino acid in the following polarizing conditions (all from eBioscience): T_H_1, anti-IL-4 (clone 11B11, 10 µg/ml) and 10 ng/ml mouse recombinant IL-12 p70; T_H_2, anti-IL-12 p40 (clone C17.8, 10 µg/ml), anti-IFN-γ (clone R4-6A2, 10 µg/ml), and 5 ng/ml mouse recombinant IL-4; T_H_17, anti-IL-4, anti-IFN-γ (clone R4-6A2, 10 µg/ml), 5 ng/ml mouse recombinant IL-6, 2.5 ng/ml recombinant IL-23, and 1.25 ng/ml recombinant TGF-β; regulatory T cell (Treg), anti-IFN-γ, 10 ng/ml TGF-β, and 10 ng/ml recombinant IL-2. Following 5 days of culture, cells were re-stimulated with 10 ng/ml phorbol 12-myristate 13-acetate (P1585, Sigma), 1 µg/ml ionomycin (I0634, Sigma) reconstituted in ethanol, and GolgiPlug (51-2301KZ, BD Biosciences) for 4 h. Cytokine production and transcription factor expression were measured by flow cytometry.

### T Cell Adoptive Transfer

CD4^+^ T cells were isolated from the spleens and peripheral lymph nodes of *Asl*^flox/+^;P25;CD45^.1/.2^ and *Asl*^flox/flox^;P25;Tie2-cre;CD45^.2/.2^ mice by magnetic sorting with the CD4 isolation kit (130-104-454, Miltenyi Biotec). Following CFSE staining, 1 × 10^6^ cells from each mouse were co-injected at an equal (i.e., 50:50) ratio into C57Bl/6;CD45^.1/.1^ mice i.v. One day following transfer, mice were infected with approximately 5 × 10^6^ *M. bovis* BCG and euthanized at the indicated time points for T cell analysis. mLNs were processed into a single cell suspension and stained for flow cytometry analysis.

### Flow Cytometry

In polarization and *in vivo* experiments, cells were first incubated with a fixable viability dye (65-0865-14, eBioscience) at 1:1,000 dilution in FACS buffer (PBS containing 1% bovine calf serum) for 10 min at room temperature. Samples were blocked using 5% normal mouse serum in FACS buffer for 10 min at 4°C. Cells were stained with antibodies for 30 min at 4°C at 1:200 dilution in FACS buffer (all from eBioscience): CD3 (clone 17A2), CD4 (clone GK1.5), CD45.1 (clone A20), and CD45.2 (clone 104). Following staining, cells were washed and fixed with Foxp3/Transcription Factor Fixation/Permeabilization buffer (00-5521, eBioscience), then stained for intracellular proteins in 1× permeabilization buffer for 30 min at 4°C. The following antibodies were used at 1:100 dilution: IFN-γ (clone XMG1.2), IL-17A (clone eBio17B7), and Foxp3 (clone FJK-16s). Data were acquired using a BD FACSCanto flow cytometer and analyzed with FlowJo software.

### Protein Analysis

Protein RIPA lysates were separated by Tris–HCl buffered 4–15% gradient SDS-PAGE, followed by transfer to Protran membranes. Membranes were analyzed by Ponceau S staining and then blocked in 3% milk in TBS plus 0.05% tween, followed by immunoblot to detect Ass1 (ab175607, Abcam), Asl (PA5-22300, Thermo Scientific), and Grb2 (610112, BD Biosciences).

### ELISA

Supernatants collected from T_H_2-polarized cell cultures were incubated overnight with plate-bound α-IL-13 capture antibody (1 µg/ml, clone eBio13A) to allow binding of synthesized IL-13. Plates were then incubated with biotinylated α-IL-13 detection antibody (500 ng/ml, clone eBio1316H) for 30 min, followed by incubation with streptavidin–horseradish peroxidase (E2886, Sigma-Aldrich). Color change of TMB substrate (34021, Thermo Scientific) stopped with 10% phosphoric acid was determined by analyzing the absorbance at 450 nm to quantify IL-13 concentration.

### Calculations and Statistics

Error bars are the SD or SEM, as described. Data were analyzed for statistical significance by Student’s *t*-test or two-way ANOVA and *p*-values are represented as *p < 0.05, **p < 0.01, and ***p < 0.001.

## Results

### l-Citrulline Rescues T Cell Proliferation and Differentiation in l-Arginine Deplete Microenvironments

**Figure 1:**
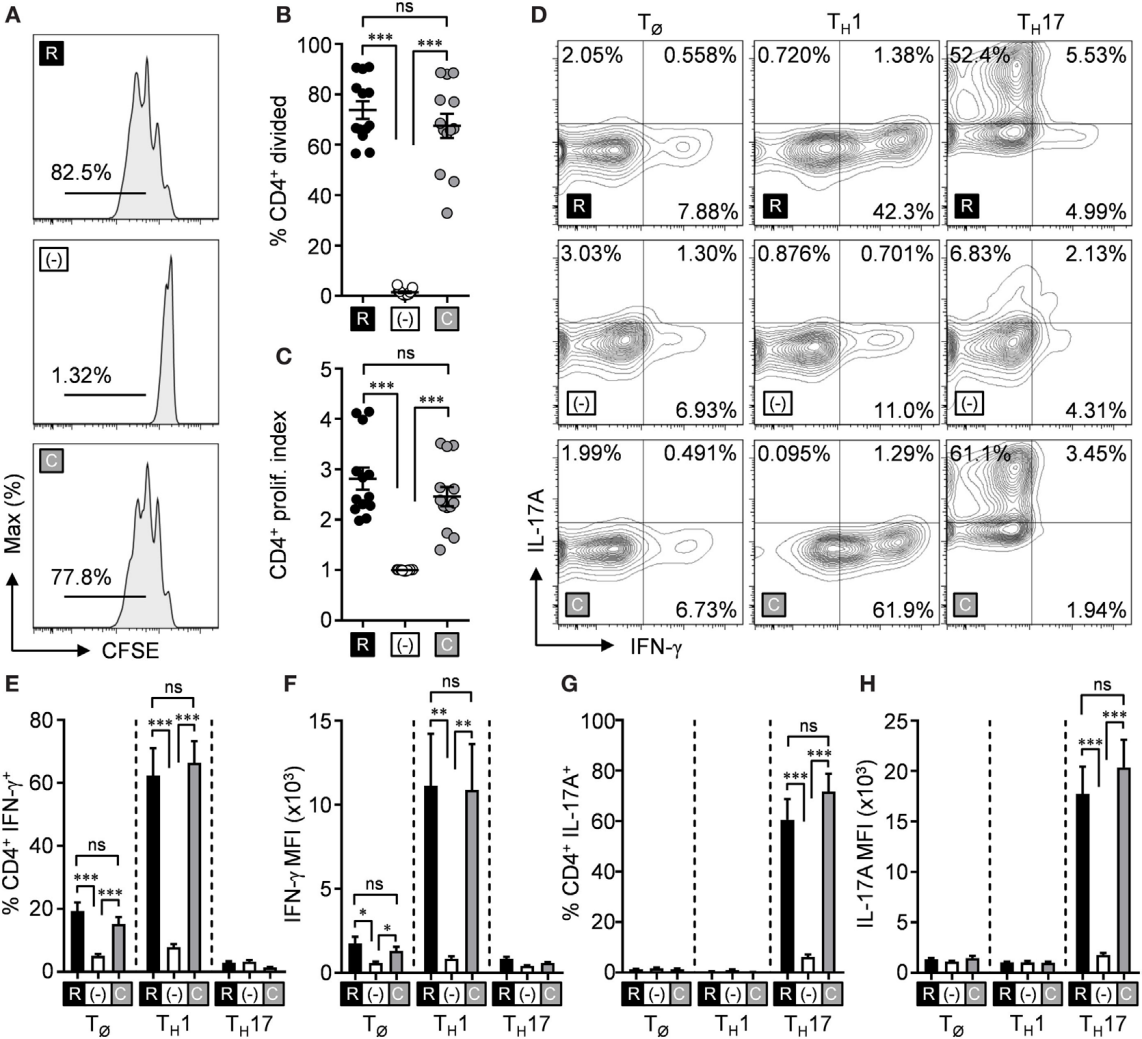
l-citrulline rescues T cell functions in the absence of l-arginine. **(A–C)** Lymphocytes from wild-type mice (C57Bl/6 background) were isolated and stained with carboxyfluorescein succinimidyl ester (CFSE). Cells were stimulated with α-CD3 and α-CD28 in R-free RPMI supplemented with 1 mM l-arginine (black), 1 mM l-citrulline (gray), or neither amino acid added (white) for 72 h. CFSE dilution of CD3^+^CD4^+^ T cells was analyzed by flow cytometry. Data are displayed as representative histograms **(A)**, mean percent of divided cells **(B)**, and proliferation index **(C)** as defined in the methods. Data are combined from four experiments. **(D–H)** Lymphocytes from wild-type mice were polarized under T_Ø_, T_H_1, or T_H_17 polarizing conditions for 5 days in the indicated culture conditions (see Materials and Methods). Following restimulation, cells were stained for intracellular cytokines and analyzed by flow cytometry. Cytokine-producing cells are represented in the graphs by mean frequency of single-positive, cytokine-expressing cells **(E,G)**, and mean fluorescence intensity of the indicated cytokine **(F,H)**. Data are combined from three experiments. Error bars, SEM. *p < 0.05, **p < 0.01, ***p < 0.001 by Student’s *t*-test.

The necessity for l-arginine to promote T cell function is well established (10, 11, 14–16). Most approaches have utilized l-arginine deficiency methods (e.g., l-arginine-free cell culture media, exogenous and/or endogenous arginase activity, etc.) to make this connection. Less focus, however, has been directed toward the impact of l-arginine synthesis (from its precursor, l-citrulline) within T cells, and how this process affects T cell effector functions. As a preliminary step to determine if l-citrulline could be utilized to promote T cell functions, we assessed CD4^+^ T cell proliferation in l-arginine-free RPMI 1640 (R-free RPMI) supplemented with l-arginine, l-citrulline, or neither amino acid. Paralleling previous work (11, 14, 25, 27), T cell proliferation was absent in l-arginine-deficient media compared with that supplemented with l-arginine (Figures 1A–C). When l-citrulline was provided, T cells restored their proliferative ability to the same degree as l-arginine-cultured cells (Figures 1A–C), indicating l-citrulline can rescue T cell proliferation when l-arginine is unavailable. These phenomena are not limited to CD4^+^ T cells, as l-citrulline also promotes CD8^+^ T cell proliferation (Figures S1A–C in Supplementary Material).

We next sought to address the necessity of these amino acids during T cell polarization—a crucial event in driving appropriate effector subsets and subsequent cytokine release essential for cell-mediated immunity. We first analyzed inflammatory cytokine production from CD4^+^ T cells cultured in non-polarizing conditions (T_Ø_), T_H_1, T_H_2, T_H_17, or Treg-polarizing conditions in R-free RPMI with l-arginine, l-citrulline, or neither amino acid added. Analogous to proliferation blockade, we observed the production of IFN-γ by T_H_1 and IL-17A by T_H_17 cells was nearly eliminated when l-arginine was absent (Figures 1D–H). Furthermore, both T_H_1 cells and T_H_17 cells regained the capacity to synthesize cytokines upon l-citrulline addition (Figures 1D–H). IL-13 production by T_H_2-polarized cells also was rescued by l-citrulline in l-arginine-deficient culture (Figure S2A in Supplementary Material). Differentiation of Treg cells, as defined by Foxp3 expression, remained unaltered in the differing amino acid conditions (Figure S3 in Supplementary Material). These data support the need for l-citrulline for T cells to maintain immunological functions when the exogenous l-arginine pool is limited.

### l-Arginine Synthesis from l-Citrulline Is Required to Rescue T Cell Effector Functions

**Figure 2:**
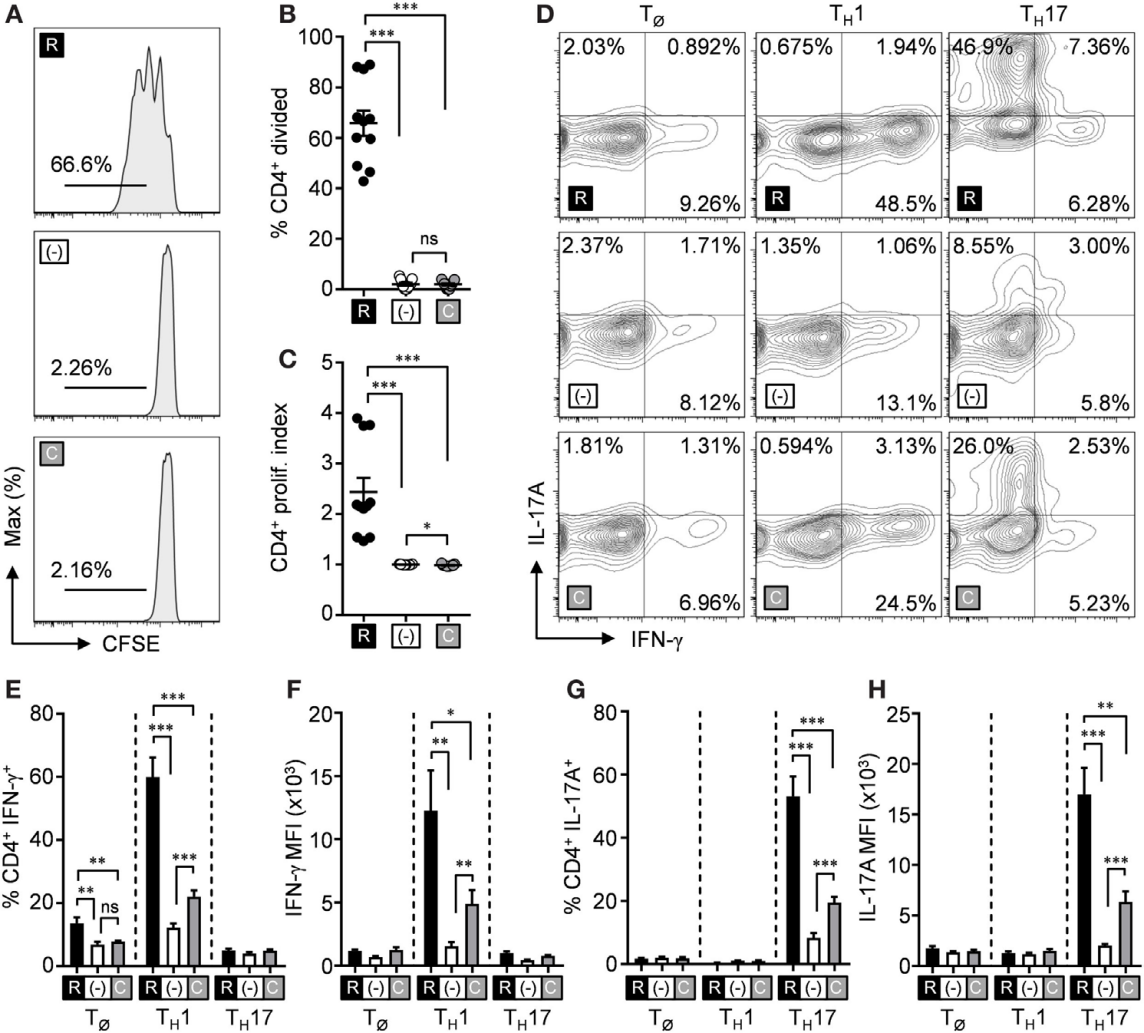
l-arginine synthesis is necessary for l-citrulline-mediated T cell functions. **(A–C)** Lymphocytes from *Asl*^flox/flox^;Tie2-cre mice were isolated and stained with carboxyfluorescein succinimidyl ester (CFSE). Cells were stimulated with α-CD3 and α-CD28 in R-free RPMI supplemented with 1 mM l-arginine (black), 1 mM l-citrulline (gray), or neither amino acid added (white) for 72 h. CFSE dilution of CD3^+^CD4^+^ T cells was analyzed by flow cytometry. Data are displayed as representative histograms **(A)**, mean percent of divided cells **(B)**, and proliferation index **(C)** as defined in the methods. Data are combined from four experiments. **(D–H)** Lymphocytes from *Asl*^flox/flox^;Tie2-cre mice were polarized under T_Ø_, T_H_1, or T_H_17 polarizing conditions for 5 days in the indicated culture conditions (see Materials and Methods). Following restimulation, cells were stained for intracellular cytokines and analyzed by flow cytometry. Cytokine-producing cells are represented in the graphs by single-positive, mean frequency of cytokine-expressing cells **(E, G)**, and mean fluorescence intensity of the indicated cytokine **(F, H)**. Data are combined from three experiments. Error bars, SEM. *p < 0.05, **p < 0.01, ***p < 0.001 by Student’s *t*-test.

l-citrulline is a non-canonical amino acid that is solely metabolized into l-arginine through the sequential activities of Ass1 and Asl, leading us to hypothesize that l-citrulline mediates T cell activity by providing a pool of synthesized l-arginine. To challenge this, lymphocytes from *Asl*^flox/flox^;Tie2-cre mice (*Asl*^ΔHEM^), that are unable to synthesize l-arginine from l-citrulline, were cultured as outlined above in Figure 1. Loss of Asl was verified in *Asl*^ΔHEM^ cells by immunoblot, while Ass1 protein expression remained unaffected (Figure S4 in Supplementary Material). Like wild-type T cells, we observed *Asl*^ΔHEM^ CD4^+^ T cells divided when provided l-arginine. However, both CD4^+^ (Figures 2A–C) or CD8^+^ T cells (Figures S1D–F in Supplementary Material) cultured in l-citrulline were unable to proliferate. When these CD4^+^ *Asl*^ΔHEM^ T cells were polarized and cytokine expression was measured, l-arginine again mediated both IFN-γ and IL-17A production, which was lost in l-arginine-deficient conditions (Figures 2D–H). Unlike wild-type cells, *Asl*^ΔHEM^ T cells provided l-citrulline significantly decreased synthesis of IFN-γ and IL-17A. This reduction, however, did not mirror the complete loss of cytokine in the R-free RPMI condition, suggesting an advantage for l-citrulline in cytokine production—but not proliferation—that is independent of l-arginine synthesis. When measured by ELISA, *Asl*^ΔHEM^ T_H_2 cells were unable to produce IL-13 in l-citrulline media (Figure S2B in Supplementary Material). Altogether, these data reveal functional advantages for l-arginine synthesis from l-citrulline by T cells in a microenvironment where extracellular l-arginine is insufficient.

### l-Citrulline Overcomes Arginase-Mediated Mycobacterial-Specific T Cell Suppression

Thus far, our data show the necessity of l-citrulline when l-arginine is artificially limited, yet l-arginine can also be reduced by the immunoregulatory properties of myeloid Arg1. Arg1 is upregulated in myeloid cells—including macrophages, neutrophils, and myeloid-derived suppressor cells—in response to various conditions, including infections, cancer, and pregnancy (17, 28, 29). Arg1 activity sequesters l-arginine from the microenvironment, creating local competition for the remaining l-arginine pool by surrounding cells. Arg1 activity alone suppresses T cells, and causes T cell hyporesponsiveness (11, 14, 18). This potentially could be detrimental during mycobacterial disease, where anti-mycobacterial T cells might be suppressed by Arg1^+^ myeloid cells, resulting in a blunted host response to infection.

**Figure 3:**
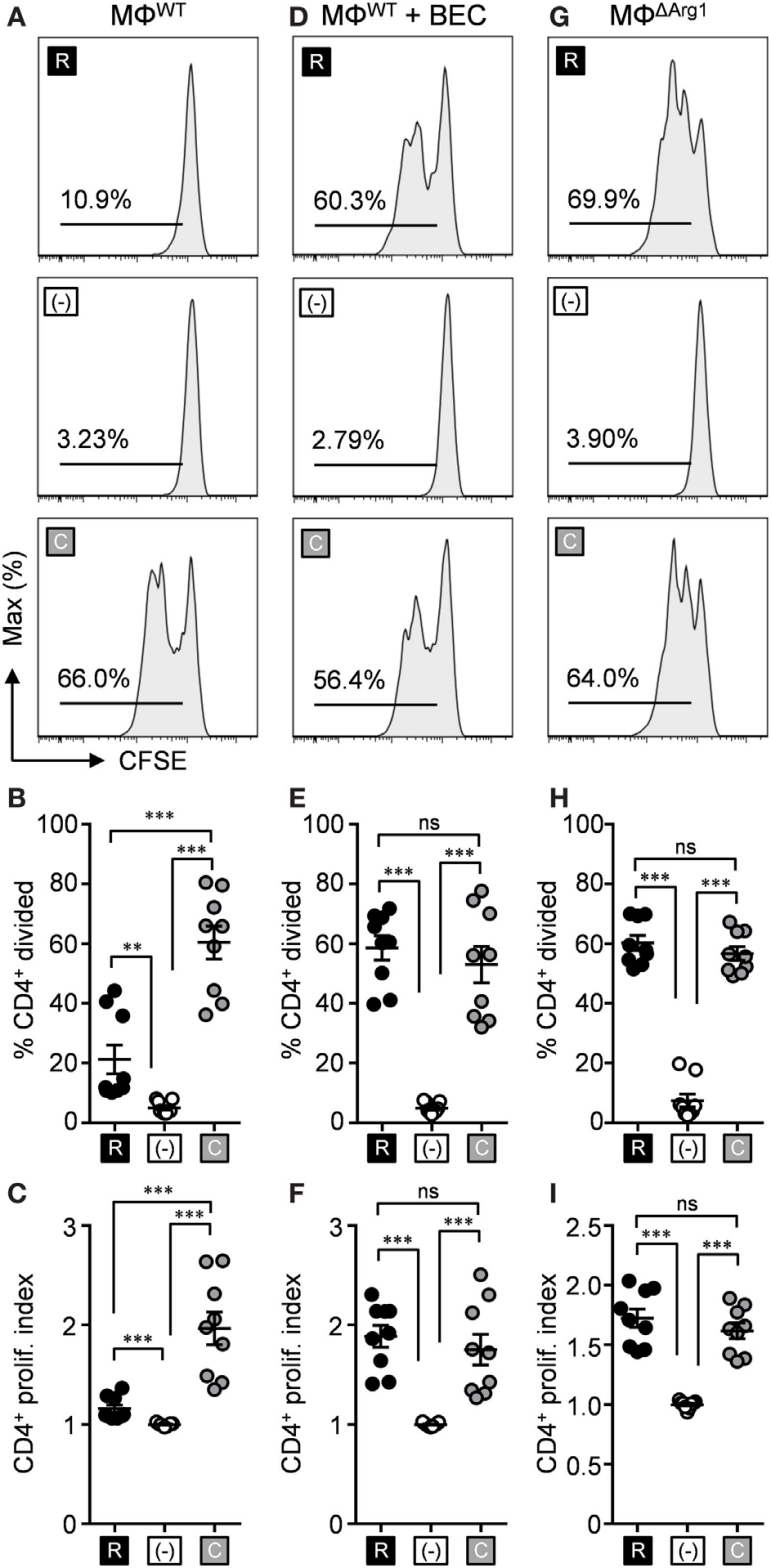
l-citrulline metabolism prevents arginase-mediated suppression of anti-mycobacterial T cells. Carboxyfluorescein succinimidyl ester (CFSE)-labeled lymphocytes from P25 mice were cocultured with HK-BCG-pulsed wild-type macrophages (MΦ^WT^) **(A–C)**, HK-BCG-pulsed wild-type macrophages treated with BEC **(D–F)**, or HK-BCG-pulsed *Arg1*^flox/flox^;Tie2-cre macrophages (MΦ^ΔArg1^) **(G–I)**. Cocultures were incubated for 72 h with 1 mM l-arginine (black), 1 mM l-citrulline (gray), or neither amino acid (white). CFSE dilution of CD3^+^CD4^+^ T cells was analyzed by flow cytometry. Data are displayed as representative histograms **(A,D,G)**, mean percent of divided cells **(B,E,H)**, and proliferation index **(C,F,I)** as defined in Section “Materials and Methods.” Data are combined from three experiments. Error bars, SEM. **p < 0.01, ***p < 0.001 according to Student’s *t*-test.

Since our data demonstrate l-citrulline as an alternative source of l-arginine, we next asked if l-citrulline would confer an advantage for T cell function in the face of arginase-mediated suppression during mycobacterial infection. We modeled amino acid competition *in vitro* to analyze how l-citrulline affects mycobacterium-specific T cell proliferation. Thioglycollate-elicited peritoneal macrophages were primed with heat-killed *M. bovis* BCG and cocultured with transgenic P25 CD4^+^ T cells, which are specific for antigen 85b of mycobacteria species. This enabled us to model the interaction of mycobacteria-containing macrophages in the lung with mycobacterial antigen-specific T cells. When P25 T cells were cultured with BCG-primed macrophages, they displayed blunted proliferation when cultured in l-arginine when compared with l-citrulline (Figures 3A–C). This was not due to a failure of CD4^+^ T cells to utilize exogenous l-arginine, as they showed a similar proliferative capacity in response to p25 peptide when cultured in l-arginine or l-citrulline, when compared with the polyclonal T cells described in Figure 1 (Figures S5A–C in Supplementary Material). We reasoned that increased Arg1 activity in BCG-primed macrophages accounted for the failure of T cells to proliferate. When we blocked Arg1 activity, either by BEC administration, or using Arg1-deficient macrophages from *Arg1*^flox/flox^;Tie2-cre mice (MΦ^ΔArg1^), we observed a restoration in P25 T cell proliferation in the presence of l-arginine (Figures 3D–I). In some experiments when Arg1 was induced in macrophages by IL-4 and IL-10 prestimulation (28), we again observed P25 cells were unable to proliferate in conditions containing l-arginine (Figure S5 in Supplementary Material). Yet, in all conditions when provided l-citrulline, P25 T cells maintained their proliferative ability (Figure 3; Figure S5 in Supplementary Material), demonstrating that T cell l-arginine synthesis from l-citrulline not only supports T cell proliferation, but also escapes Arg1-mediated immunosuppression imposed during mycobacterial and/or cytokine stimulation.

**Figure 4:**
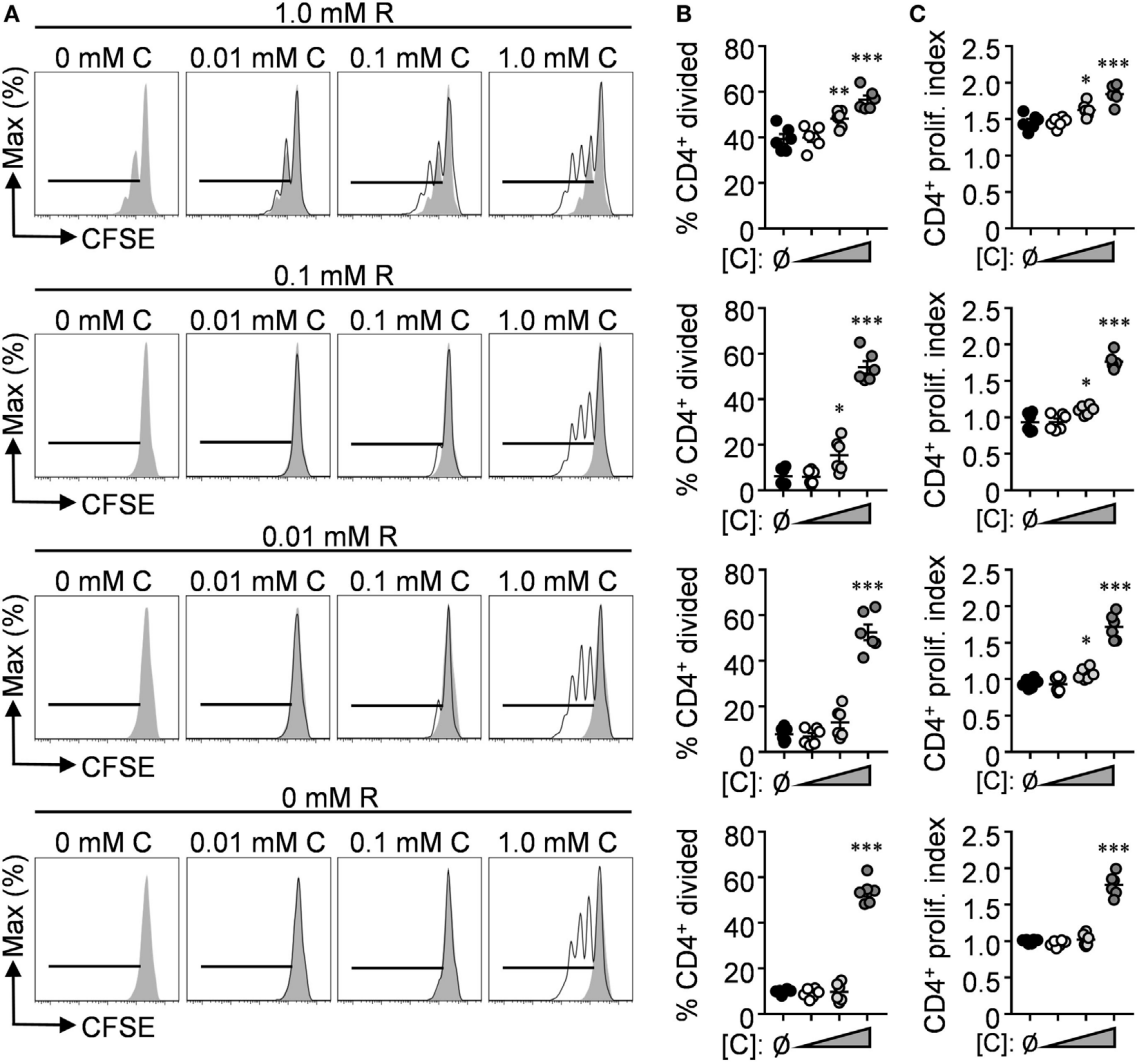
l-citrulline enhances mycobacterium-specific T cell proliferation in l-arginine-sufficient culture. **(A–C)** Carboxyfluorescein succinimidyl ester (CFSE)-labeled lymphocytes from P25 mice were cocultured with HK-BCG-pulsed wild-type macrophages for 72 h with titrating concentrations of l-arginine (**A**, gray histograms) and l-citrulline (**A**, black histograms). CFSE dilution of CD3^+^CD4^+^ T cells was analyzed by flow cytometry. Data are displayed as representative histograms **(A)**, mean percent of divided cells **(B)**, and proliferation index **(C)** as defined in the methods. Data are combined from two experiments. Error bars, SEM. *p < 0.05, **p < 0.01, ***p < 0.001 compared with no l-citrulline added according to Student’s *t*-test.

Although these findings provide insight into the potential benefits of l-citrulline over l-arginine during an anti-mycobacterial T cell response, T cells are likely to encounter much smaller concentrations of these amino acids *in vivo*. The concentrations of free l-arginine and l-citrulline in the lung microenvironment remain to be established; however, in homeostatic serum l-arginine and l-citrulline are maintained at a range of 50–250 µM, prompting us to investigate the effects of l-citrulline and l-arginine at a range of concentrations for T cell proliferation. To do so, P25 T cells were cocultured with BCG-pulsed macrophages as before, but also in titrating concentrations of l-arginine and l-citrulline. Like our previous data (Figure 3), T cell division in 1 mM l-arginine was blunted, presumably due to the arginase activity of the macrophages (Figures 4A–C). Upon addition of l-citrulline, we observed a significant increase in proliferation by T cells supplemented with 1 or 0.1 mM l-citrulline. l-citrulline supplementation also enhanced proliferation in cultures given physiological (0.1 mM), low (0.01 mM), or no l-arginine, although the beneficial effects of 0.1 mM l-citrulline waned once l-arginine availability became low. Taken altogether, these data suggest a physiologically important contribution of l-citrulline for mycobacterium-specific T cell function.

### l-Citrulline Is Necessary for T Cell Accumulation in the Mycobacteria-Infected Lung and Draining mLNs *In Vivo*

**Figure 5:**
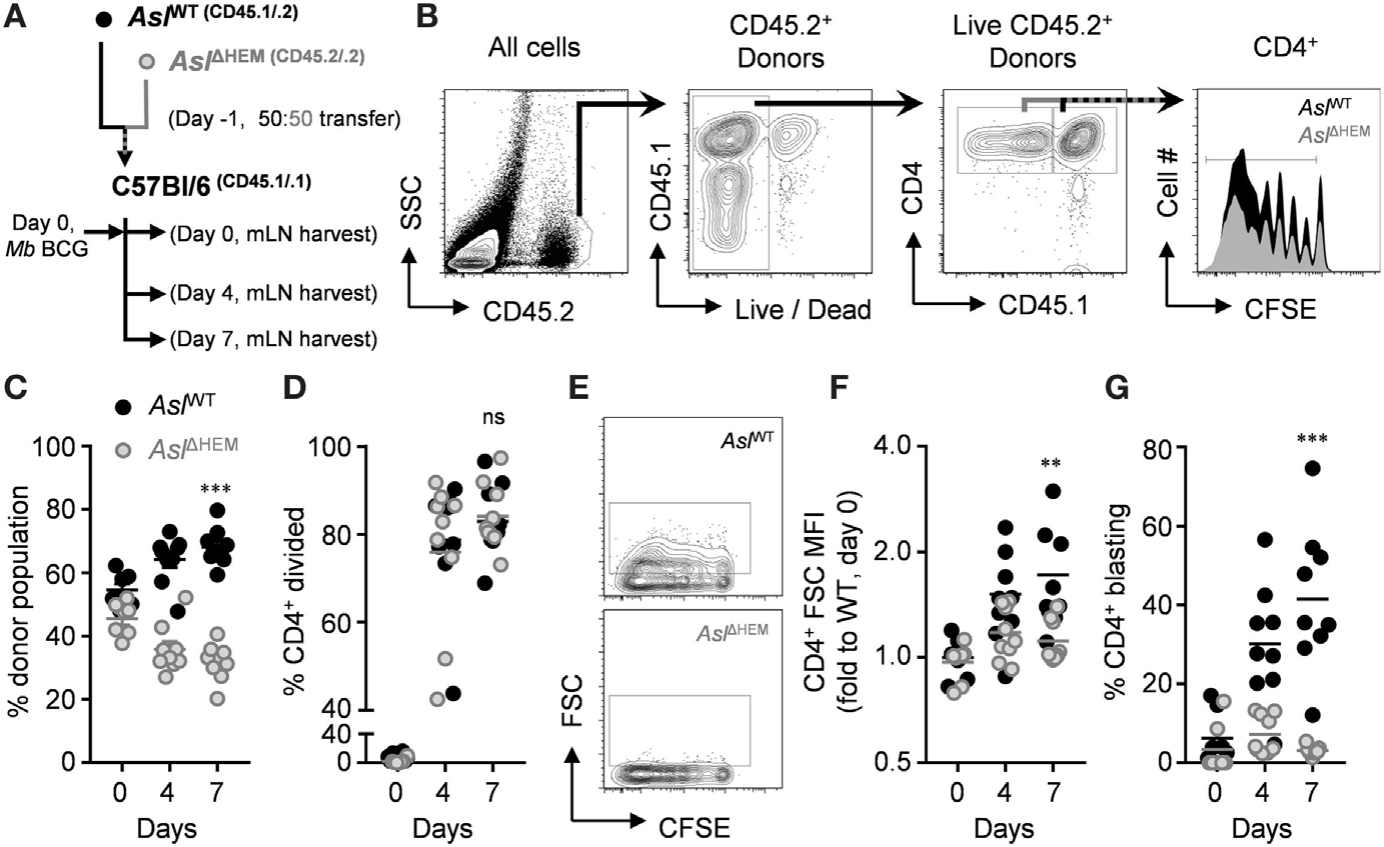
l-citrulline metabolism drives anti-mycobacterial CD4^+^ T cell blasting and accumulation in the mLN upon *M. bovis* BCG infection. **(A)** CD4^+^ T cells were collected from P25k;*Asl*^flox/flox^;Tie2-cre (*Asl*^ΔHEM^) and P25k;*Asl*^flox/flox^;(−) (*Asl*^WT^) mice and labeled with carboxyfluorescein succinimidyl ester (CFSE). Cells were mixed at a 50:50 ratio and transferred i.v. to C57Bl/6 mice (*n* ≥ 7) one day prior to *M. bovis* BCG infection (i.n., ~5 × 10^6^ colony forming units). Lung draining mLNs were collected the day after transfer from uninfected mice (day 0), and at 4 and 7 days postinfection. **(B)** Each donor population and the recipient cells were identified based on congenic CD45 variants, and proliferation of donor T cells was measured by CFSE dilution. Cells were analyzed for ratio of total donor cells **(C)**, proliferation **(D)**, FSC-calculated size **(E,F)**, and blasting frequency [**(G)**, see gated population in **(E)**]. Data are combined from two experiments. **p < 0.01, ***p < 0.001 by two-way AVOVA.

l-arginine restriction during mycobacterial disease poses a challenge for responding T cells to overcome in infected tissues. Previous data have shown that loss of Arg1 leads to improved mycobacterial host defense early, but allows T cell-mediated immunopathology in late stages of infection (21, 30), reminding us that regulating a balance of activation and inhibition is a key property during immune responses. Our data uncover that l-citrulline can replenish l-arginine for T cells, which led us to hypothesize that metabolism of this amino acid would be necessary to sustain T cell-mediated defense against mycobacterial infection *in vivo*. As a first step to address this, we asked if l-citrulline provided any advantage to T cells early during mycobacterial infection *in vivo*. CD4^+^ T cells were isolated from P25;*Asl*^flox/+^;CD45^.1/.2^ (*Asl*^WT^) and P25;*Asl*^flox/flox^;Tie2-cre;CD45^.2/.2^ (*Asl*^ΔHEM^) mice and labeled with CFSE to track proliferation. Cells were then co-transferred into C57Bl/6;CD45^.1/.1^ mice at an equal ratio (Figure 5A) in order to distinguish both donor populations from the endogenous lymphocytes (Figure 5B). Mice were then infected with *M. bovis* BCG and donor cells in the mLN were analyzed prior to infection (day 0) and 4 and 7 days postinfection. Upon analysis of the transferred cells across time, Asl-deficient P25 T cells were reduced in the lymph node compared with Asl-sufficient donor cells (Figure 5C). When CFSE dilution was quantified in these cells, we observed the *Asl*^WT^ and *Asl*^ΔHEM^ T cells possessed the same capability to divide and similar viability *in vivo* (Figure 5D; Figure S6 in Supplementary Material), suggesting the difference in frequency was not necessarily due to proliferative capacity or increased death at the time of analysis. Interestingly, Asl-deficient donor cells had reduced cell size and blasting compared with *Asl*^WT^ (Figures 5E–G; Figures S6C,E in Supplementary Material), suggesting that l-citrulline metabolism promotes a growth and accumulation advantage to mycobacterium-specific CD4^+^ T cells early during infection.

**Figure 6:**
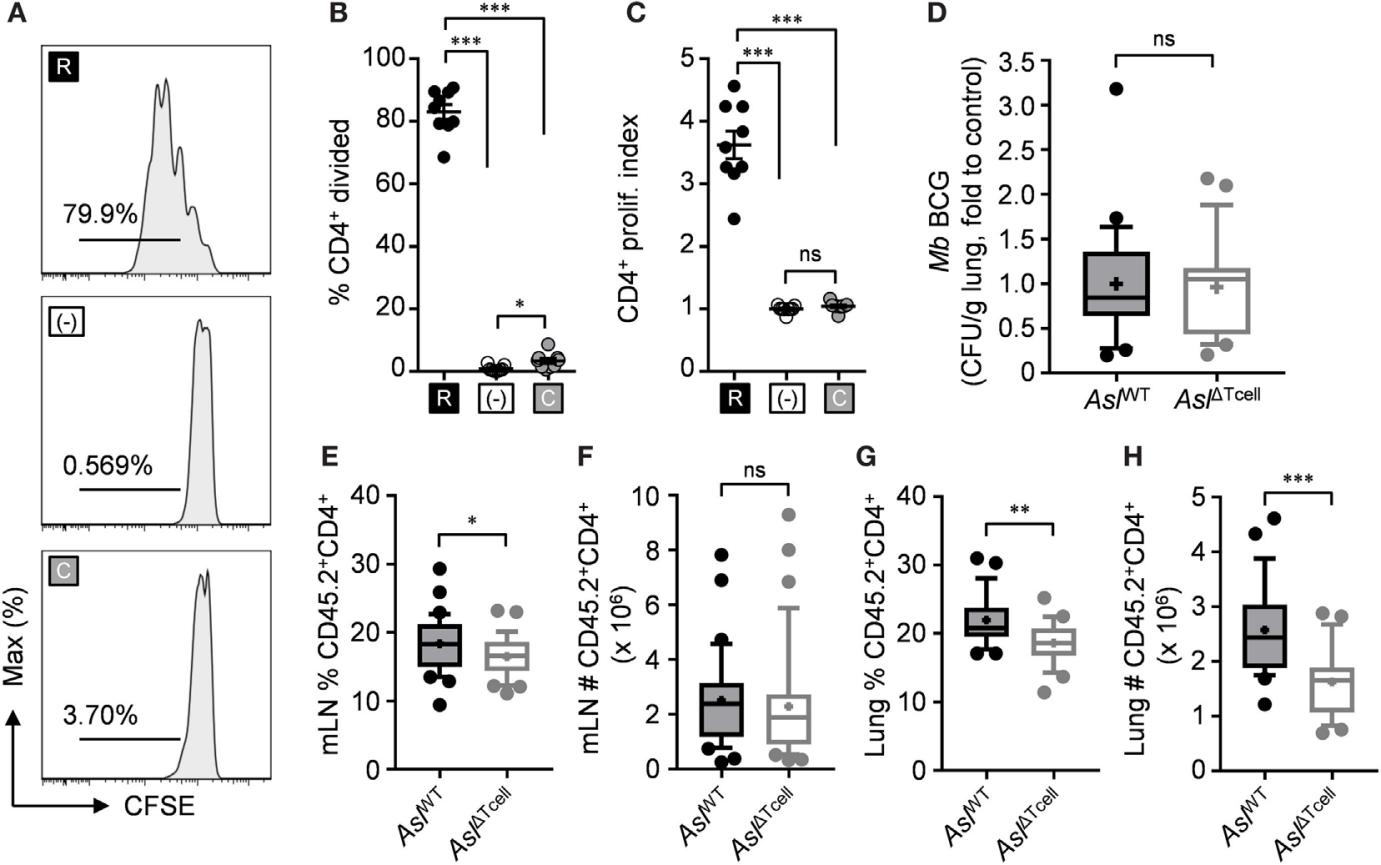
l-citrulline metabolism is necessary for CD4^+^ T cell accumulation in the mycobacterial infected lung and mediastinal lymph node *in vivo*. **(A–C)** Lymphocytes from *Asl*^flox/flox^;CD4-cre (*Asl*^ΔTcell^) were isolated and stained with carboxyfluorescein succinimidyl ester (CFSE). Cells were stimulated with α-CD3 in R-free RPMI supplemented with 1 mM l-arginine (black), 1 mM l-citrulline (gray), or neither amino acid added (white) for 72 h. CFSE dilution of CD3^+^CD4^+^ T cells was analyzed by flow cytometry. Data are displayed as representative histograms **(A)**, percent of divided cells **(B)**, and by proliferation index **(C)** as defined in the methods. Data are combined from three experiments. **(D–H)** *Asl*^flox/flox^ (*Asl*^WT^) and *Asl*^ΔTcell^ mice were infected with ~5 × 10^6^ colony forming unit *M. bovis* BCG intranasally. Eight weeks postinfection, mycobacterial burden was quantified in the lungs **(D)**. CD45.2^+^CD4^+^ cells were quantified from the mediastinal lymph node **(E,F)** and infected lung **(G,H)**. Data are combined from three experiments and are displayed as box and whisker plots (*n* ≥ 22). Error bars, SEM. *p < 0.05, **p < 0.01, ***p < 0.001 by Student’s *t*-test.

Since l-arginine synthesis provided an advantage for accumulation of transferred T cells in the draining mLN following infection, we next sought to determine if l-citrulline metabolism was necessary for endogenous anti-mycobacterial T cell accumulation and host defense during infection. To do this, we first developed a model to conditionally delete l-citrulline metabolism in the T cell compartment only. *Asl*^flox/flox^;CD4-cre (*Asl*^ΔTcell^) mice were generated to selectively delete Asl in T cells, which results in a loss of detectable protein by immunoblot in both naïve and activated T cells without affecting other hematopoietic populations (Figure S4 in Supplementary Material). To validate functional deletion of l-arginine synthesis in CD4^+^ T cells from these mice, we analyzed their proliferation in l-arginine or l-citrulline sufficient conditions *in vitro*. l-arginine supplemented culture enabled CD4^+^ *Asl*^ΔTcell^ T cells to proliferate (Figures 6A–C), to a similar extent as wild-type cells in Figure 1. However, those cultured in l-citrulline resulted in loss of proliferation, (Figures 6A–C) mirroring the *Asl*^ΔHEM^ T cells (Figure 2) and confirming that l-citrulline metabolism to l-arginine was disrupted in T cells from these mice. Subsequently, *Asl*^ΔTcell^ mice and wild-type littermate controls (*Asl*^WT^) were infected with *M. bovis* BCG to test the necessity of l-citrulline for anti-mycobacterial T cell responses. Surprisingly, upon analysis of mycobacterial burden in the lung, mice lacking T cell l-citrulline metabolism displayed no discernable differences compared with controls (Figure 6D). Similar data were found when infecting these mice with virulent *M. tuberculosis* (*Asl*^WT^ = 6.45 ± 5.13 versus *Asl*^ΔTcell^ = 6.21 ± 5.03 log_10_ CFU, Figure S7 in Supplementary Material). As such, the T cell infiltrate could be analyzed independent of the influence of pathogen burden. Similar to our adoptive transfer data, *Asl*^ΔTcell^ mice exhibited reduced frequency of CD4^+^ T cells in the mLNs, and also reduced CD4^+^ T cell accumulation in the infected lung (Figures 6E–H), providing further evidence that l-citrulline metabolism supports T cell accumulation in relevant tissues following mycobacterial infection *in vivo*.

## Discussion

Taken together, these data reveal a role for the amino acid l-citrulline during anti-mycobacterial T cell responses. Our study builds upon noteworthy findings from several groups demonstrating the potential of this amino acid to rescue proliferation of hyporesponsive T cells from human T cell lines (11, 31–33), and primary cells from human (14, 25) and mouse (25). Our data not only confirm the importance of l-citrulline on cellular division, but also extend these findings to additional aspects of T cell immunity. While l-citrulline metabolism deficiency is reported to negatively affect T cell differentiation in l-arginine culture (25), our data show *Asl*^WT^ and *Asl*^ΔHEM^ T cells are equally capable of producing cytokines in the presence of l-arginine. The previous findings were discovered in a model of citrullinemia where mouse viability was rescued by gene transfer of the *Ass1* to the liver (34). Despite rescued survival, treated mice exhibited only 4% Ass1 activity compared with wild-type controls, resulting in 2.5-fold increase in plasma ammonia. This amount of ammonia has been suggested to be toxic to circulating lymphocytes, reducing viability as well as activation potential (35), which could cause endogenous differences in T cell functions compared with our model that bypasses liver interference. The published model provides an important approach for studying systemic consequences following citrullinemia treatment, and the system we utilize here allows investigating the mechanism of l-citrulline metabolism specifically for immune cell functions.

Interestingly, our data do not show a complete loss of cytokine production when Asl-deficient T cells were cultured in l-citrulline. Asl-deficient T cells in l-citrulline mirror the l-arginine-free condition in terms of proliferation, but cytokine production persists to a higher degree than the l-arginine-starved cells. Auphan-Anezin et al. observed a similar phenomenon in CD8^+^ T cells, where IFN-γ production occurred before cellular division (36). Yet, if the reduced level of cytokine production in our CD4^+^ T cells is l-arginine independent, T cells cultured in l-arginine-free conditions would exhibit a similar level of cytokine production. If this phenomenon is independent of l-arginine synthesis from l-citrulline, we first speculate that l-citrulline may be detected directly to modulate T cell function. Recently, the sodium-coupled neutral amino acid transporter SLC38A9 has been identified to signal sufficiency of the amino acids l-arginine, l-leucine, and l-glutamine to mTORC1 (37–39), opening the possibility that l-citrulline levels may be sensed in this way to affect cellular metabolism. However, these findings were generated using non-hematopoietic and myeloid cell lines, and thus leaving the ability of l-citrulline to interact with this transporter in T cells unknown. One transport system recently identified to transport l-citrulline in human T cells is the large neutral amino acid transporter LAT-1 (13), yet signaling potential of this transporter has not been detected or identified. Second, we hypothesize that residual activity of Asl in *Asl*^ΔHEM^ T cells is driving moderate cytokine production in Asl-deficient cells despite an inability for these cells to proliferate—possibly due to different amino acid concentration thresholds necessary for one function versus the other in T cells. We did not detect Asl protein expression by immunoblot in naïve or activated T cells, however, further metabolic studies tracing l-citrulline fate in polarized T cells will help address the functional loss of Asl in this knockout model.

Our data also show expression of the inflammatory cytokines IFN-γ and IL-17A by polarized T cells can be restored in l-arginine-deficient conditions by l-citrulline metabolism. Over the last several decades, IFN-γ-producing T cells have been shown to be crucial for anti-mycobacterial immunity, providing activation to infected macrophages, and subsequent pathogen resistance (4, 7, 40). Although the role of T_H_17 cells in mycobacterial disease remains controversial in the literature, IL-17A has been implicated in protective responses against several pathogens (41–43) and a reduced frequency of T_H_17 cells is associated with active TB disease and its severity in the clinic (44, 45). In an l-arginine-restricted microenvironment, our data would suggest that l-arginine synthesis from endogenous l-citrulline could bolster inflammatory cytokine production that may otherwise be suppressed. Yet, considering IL-13 production was also rescued by l-citrulline metabolism, cytokine production *via* l-arginine synthesis may be broadly applicable across the spectrum of cell-mediated immunity.

In human TB disease, Arg1-expressing myeloid cells are found within granulomas (19), and patients with elevated type 2 cytokines, such as arginase-inducing IL-4 and IL-13, are reported to exhibit worse clinical disease outcomes (46, 47). In an Arg1-suppressive environment, our data suggest that mycobacterium-specific T cells provided with l-citrulline might maintain their inflammatory response driven by l-arginine synthesis. In addition, we found that l-citrulline endowed a proliferative advantage to these T cells in physiologically relevant concentrations (i.e., 0.1 mM). Yet we still do not know the availability of l-arginine and l-citrulline systemically or locally during infection, when l-arginine synthesis in the body becomes essential. Further analysis of amino acid concentrations during infection are necessary to determine if anti-mycobacterial T cells rely on l-citrulline to fuel optimal function. Moreover, supraphysiological l-citrulline addition resulted in greater enhancement of T cell proliferation at all l-arginine concentrations. This suggests supplemental l-citrulline could incite enhanced T cell proliferation in the face of l-arginine deprivation, warranting future studies to investigate if increasing the availability of l-citrulline to T cells *in vivo* would enhance T cell anti-mycobacterial immunity. As revealed by our *in vivo* approaches, we also find that l-citrulline metabolism promotes CD4^+^ T cell growth and accumulation in relevant sites of mycobacterial infection. Recently, CD8^+^ T cell survival and anti-tumor activity *in vivo* were shown to be enhanced by increased intracellular l-arginine levels (48), suggesting the reduction in CD4^+^ T cell accumulation in the mLN and lungs of Asl-deficient mice may be tied to decreased survival. The mechanism by which l-citrulline metabolism affects CD4^+^ T cell accumulation and/or growth during infection remains to be further examined with detailed longitudinal studies.

Previous reports have demonstrated that T cell influx into the lung occurs between 2 and 4 weeks postmycobacterial infection in mice (40, 49), leading us to first question if l-citrulline metabolism is necessary for the early response of CD4^+^ T cells. Despite the interesting disparity in CD4^+^ T cell numbers in the lung, we did not observe differences in mycobacterial burden between *Asl*^WT^ and *Asl*^ΔTcell^ mice. However, this strengthens our findings that the effect of l-citrulline metabolism on driving CD4^+^ T cell accumulation is T cell-intrinsic and not due to differences in antigen abundance in the mice. We hypothesize that the responsibility of l-citrulline metabolism to initially drive T cell accumulation may affect pathogen survival later in infection; however, further studies are needed to elucidate the kinetics of T cell accumulation and maintenance and their subsequent consequences on infection at later time points. An important aspect of directly infecting the *Asl*^ΔTcell^ mice is a possibility that “less fit” T cells could be repopulated during hematopoiesis—which cannot occur in our adoptive transfer approach (Figure 5). This is one possible reason for the disparity in the results from these two approaches. Additionally, anti-mycobacterial host defense in *Asl*^ΔTcell^ mice could be mediated by inefficient deletion of *Asl* in the pan T cell population and subsequent outgrowth of Asl^+^ clones. Future *in vivo* infection experiments with these mice will assess *Asl* expression and the capacity of l-citrulline metabolism within responding T cells.

In addition to mycobacterial disease, T cells are manipulated *via* l-arginine metabolism by myeloid cells in other conditions, including various cancers. Myeloid cells expressing elevated Arg1 from a lung carcinoma model blunt T cell function *via* l-arginine sequestration and facilitate tumor escape (50), and CD8^+^ T cells supplemented with l-arginine mount greater anti-tumor responses than untreated controls (48). Considering the competition for l-arginine by a variety of cells in the tumor microenvironment, targeting the l-citrulline metabolic pathway may also serve to promote anti-tumor T cell immunity. Conversely, in diseases such as autoimmunity or graft-versus-host disease, T cell inflammation needs to be blunted, not exacerbated (51, 52). Many effective therapies for these conditions rely on global immune suppression, rendering patients highly susceptible to other complications, such as infection (53, 54). The l-citrulline metabolic machinery may be a more innocuous target for immunomodulation to reduce inflammatory T cell functions by blocking intrinsic synthesis of l-arginine. Further, our data demonstrate polarization of regulatory CD4^+^ Foxp3^+^ cells is independent of l-arginine and l-citrulline conditions. Although the consequences of l-citrulline metabolism on the functional capacity of Treg cells still remains undefined, our data suggest that targeting l-citrulline metabolism to dampen inflammatory T cells could leave Treg polarization intact, further assisting therapeutic potential.

l-citrulline metabolism may also be linked to other metabolic processes in T cells. The energy acquiring metabolic profile of T cells is known to change with metabolic demands of different T cell phenotypes. For example, activated T cells must switch from mainly oxidative phosphorylation in their naïve state to glycolytic pathways, and then transition to fatty acid oxidation or back to oxidative phosphorylation when adapting a regulatory phenotype or memory recall (55). Recently, Geiger et al. found that increased l-arginine in culture promotes CD8^+^ T cell oxidative phosphorylation and cell survival *in vivo* (48). l-citrulline metabolism consumes and produces byproducts involved with the TCA cycle (22) and our data here suggest a role for l-citrulline metabolism in CD4^+^ T cell survival. Whether l-citrulline metabolism influences metabolic programing and effector phenotypes in CD4^+^ T cells remains to be elucidated.

In summary, our data demonstrate the importance of l-arginine synthesis for CD4^+^ T cell function. As our previous work helped to identify the necessity of l-citrulline metabolism within the hematopoietic lineage for anti-mycobacterial immunity, these data suggest one mechanism of this phenomenon could be driving the accumulation of anti-mycobacterial CD4^+^ T cells—which could have important ramifications on therapy and/or novel vaccine design. Moreover, this work warrants further investigation into the potential for l-citrulline modulating T cells in other disorders, and its downstream effects on other interacting immune populations during disease.

## Ethics Statement

All procedures were approved by the CCHMC Institutional Animal Care and Use Committee, protocol number 2013-0176 adhering to the Guide for the Care and Use of Laboratory Animals.

## Author Contributions

Conceptualization (SL and JQ), methodology (all authors), investigation (SL, MM, SS, JM, AH, and JQ), resources (IL, SW, and JQ), writing—original draft (SL and JQ), writing—review and editing (all authors), supervision (IL, SW, JT, and JQ), and funding acquisition (SL and JQ).

## Conflict of Interest Statement

The authors declare that the research was conducted in the absence of any commercial or financial relationships that could be construed as a potential conflict of interest.
