# Supplementary material for: l-Citrulline Metabolism in Mice Augments CD4+ T Cell Proliferation and Cytokine Production In Vitro, and Accumulation in the Mycobacteria-Infected Lung
Source: Front Immunol. 2017 Nov 16;8:1561. doi: 10.3389/fimmu.2017.01561 (PMC5696333; doi:10.3389/fimmu.2017.01561)
Supplement: Supplementary file 1 [file presentation_1.pdf]

frontiers

Supplementary Material

L-citrulline metabolism in mice augments CD4$^+$ T cell proliferation and cytokine production *in vitro*, and accumulation in the mycobacteria-infected lung

Shannon M. Lange[1, 6], Melanie C. McKell[1, 6], Stephanie M. Schmidt[1], Austin P. Hossfeld[2], Vandana Chaturvedi[3], Jeremy M. Kinder[3], Jaclyn W. McAlees[4], Ian P. Lewkowich[4], Sing Sing Way[3], Joanne Turner[2,5], and Joseph E. Qualls[1,*]

[1]Laboratory of Dr. Joseph E. Qualls, Division of Infectious Diseases, Department of Pediatrics, Cincinnati Children's Hospital Medical Center, Cincinnati OH, USA.
[2]Laboratory of Dr. Joanne Turner, Department of Microbial Infection & Immunity, College of Medicine, The Ohio State University, Columbus OH, USA.
[3]Laboratory of Dr. Sing Sing Way, Division of Infectious Diseases, Department of Pediatrics, Cincinnati Children's Hospital Medical Center, Cincinnati OH, USA.
[4]Laboratory of Dr. Ian P. Lewkowich, Division of Immunobiology, Department of Pediatrics, Cincinnati Children's Hospital Medical Center, Cincinnati OH, USA.
[5]Texas Biomedical Research Institute, San Antonio TX, USA.
[6]Immunology Graduate Program, University of Cincinnati / Cincinnati Children's Hospital Medical Center, Cincinnati OH, USA.

***Correspondence:**
Joseph E. Qualls, Department of Pediatrics, Division of Infectious Diseases, MLC 7017, Cincinnati Children's Hospital Medical Center, 3333 Burnet Ave, Cincinnati, OH 45229. Email: joseph.qualls@cchmc.org,513-636-9102 (Telephone), 513-636-7039 (Fax).

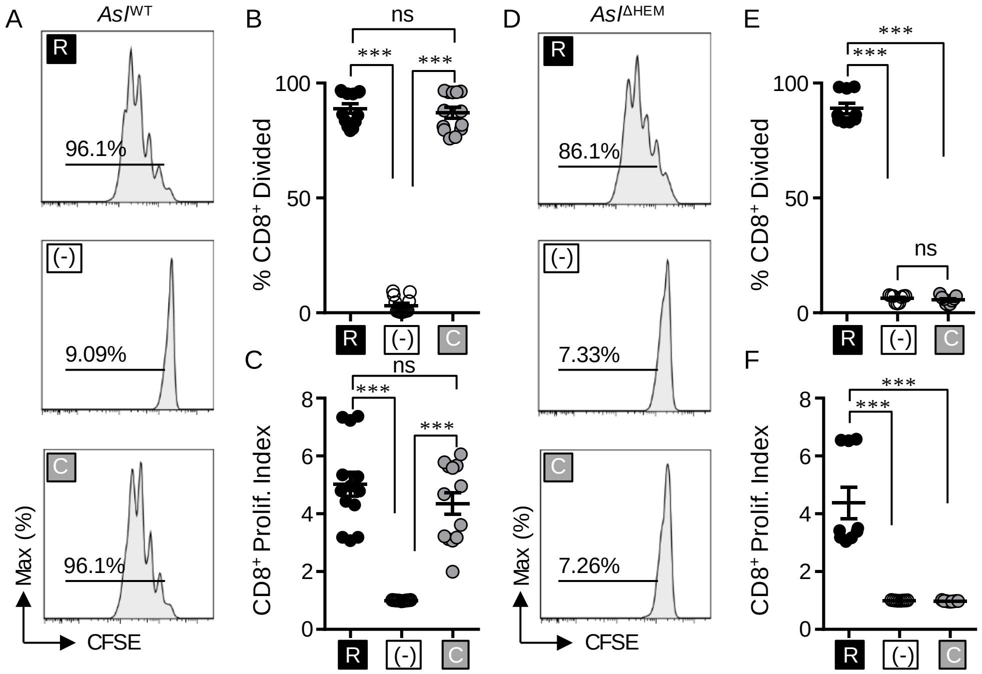

Figure S1. CD8⁺ T cells from *Asl*ΔHEM mice cannot metabolize L-citrulline to drive proliferation. CFSE-labeled lymphocytes from *Asl*flox/flox;Tie2-cre (*Asl*ΔHEM) mice (**D-F**) or wild-type controls (**A-C**) were stimulated with α-CD3/28 in R-free RPMI supplemented with 1mM L-arginine (black), 1mM L-citrulline (gray), or neither amino acid added (white) for 72 hours and analyzed by flow cytometry. Data are displayed as representative histograms (A,D), percent of divided cells (B,E), and by proliferation index as defined in the methods (C,F). Data are from at least three experiments combined. Error bars, SEM. ***p<0.001 by Student's T test.

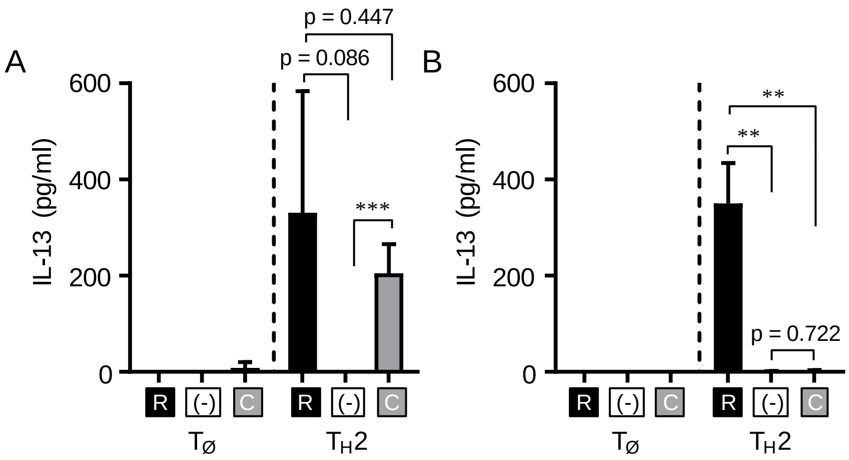

Figure S2. L-arginine synthesis is necessary for TH2 cytokine production in L-citrulline. Splenocytes from *Asl*^{WT} (**A**) and *Asl*$^{\Delta HEM}$ (**B**) mice were stimulated and polarized under TØ or TH2 polarizing conditions for 5 days with α-CD3 in the indicated culture conditions (see Methods and Materials). Supernatants were collected and IL-13 production was analyzed by ELISA. Data are from one experiment. Error bars, SD. ** $p < 0.01$, *** $p < 0.001$ by Student’s t test.

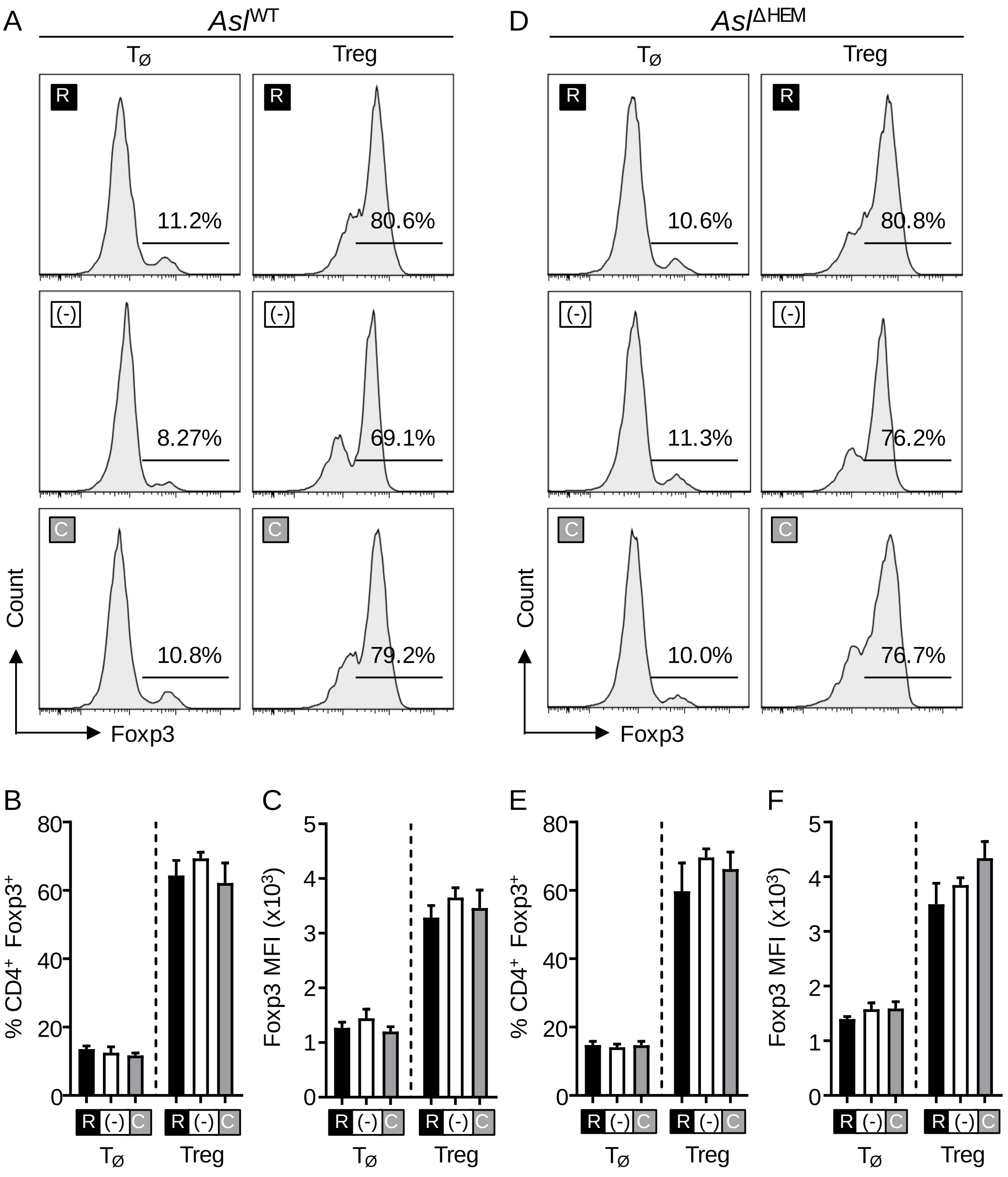

Figure S3. Regulatory T cell induction *in vitro* is independent of L-arginine/L-citrulline availability. Lymphocytes from *Asl*^{WT} (**A-C**) or *Asl*$^{\Delta HEM}$ mice (**D-F**) were polarized under Tø or Treg polarizing conditions with α-CD3 for 5 days in 1mM L-arginine (black), 1mM L-citrulline (gray), or neither amino acid (white). Following restimulation, cells were stained for Foxp3 expression and analyzed by flow cytometry. Live CD4^{+}Foxp3^{+} cells are represented in the graphs by frequency of Foxp3 expression (B, E) and mean fluorescence intensity (C, F). Data are displayed as mean values + SEM. Data are combined from three experiments.

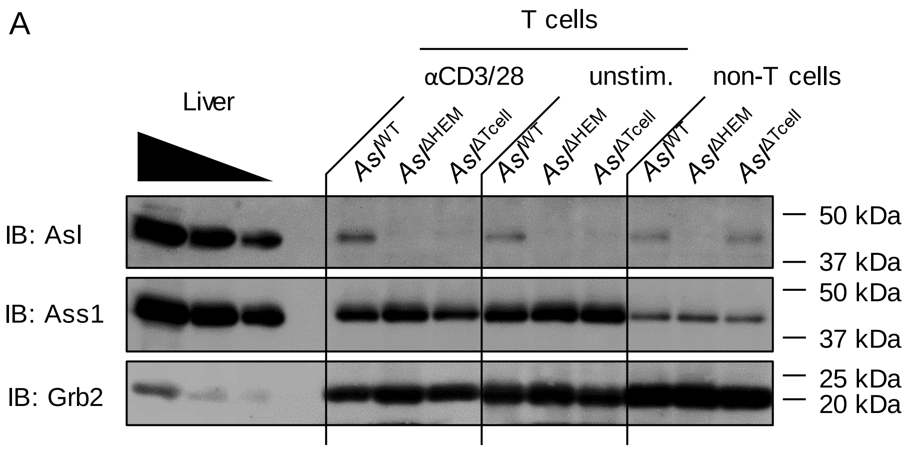

Figure S4. Deletion of Asl in hematpoietic and T cell compartments within conditional knockout mouse strains. (**A**) Pan T cells were magnetically purified from spleens and lymph nodes of Asl^{WT}, $Asl^{\Delta HEM}$, and $Asl^{\Delta Tcell}$ mice. The non-T cell fraction was also analyzed as a control. T cells were lysed with RIPA buffer immediately or following 72 hour stimulation with α-CD3/28 in C-RPMI. Protein lysates were collected and run on SDS-PAGE prior to immunoblotting with anti-Asl, anti-Ass-1, and anti-Grb2 (loading control) antibodies. Titrated liver lysates were run as a control for Asl and Ass1 protein. Data are representative of two independent immunoblots analyzing lysates from distinct mice. Data are from one experiment.

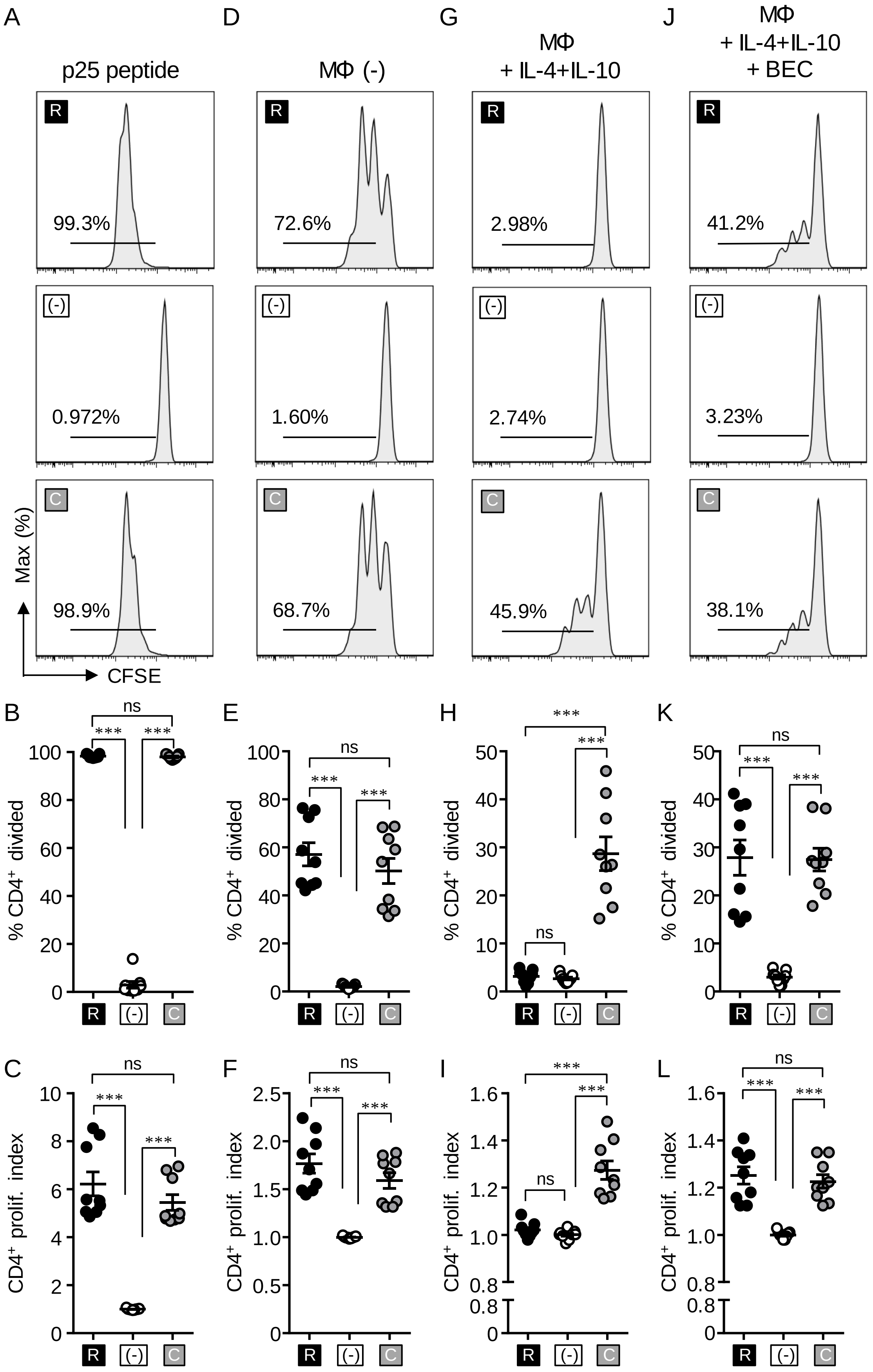
A
p25 peptide
D
MΦ (-)
G
MΦ + IL-4+IL-10
J
MΦ + IL-4+IL-10 + BEC
R
(-)
C
99.3%
0.972%
98.9%
72.6%
1.60%
68.7%
2.98%
2.74%
45.9%
41.2%
3.23%
38.1%
Max (%)
CFSE
B
E
H
K
% CD4+ divided
C
F
I
L
CD4+ prolif. index
ns
***

Figure S5. L-citrulline metabolism prevents induced arginase-mediated suppression of anti-mycobacterial T cells. CFSE-labeled lymphocytes from P25 mice were cocultured with macrophages + p25 peptide (**A-C**), HK-BCG-pulsed macrophages (**D-F**), HK-BCG-pulsed macrophages prestimulated with IL-4 and IL-10 (**G-I**), or HK-BCG-pulsed macrophages prestimulated with IL-4 and IL-10 and treated with BEC (**J-L**). Cocultures were incubated for 72 hours in media containing 1 mM L-arginine (black), 1 mM L-citrulline (gray), or neither amino acid (white). CFSE dilution of $CD3^{+}CD4^{+}$ T cells was analyzed by flow cytometry. Data are displayed as representative histograms (A, D, G, J), mean percent of divided cells (B, E, H, K), and proliferation index (C, F, I, L) as defined in the methods. Data are combined from three experiments. Error bars, SEM. ***$p<0.001$ according to Student's t test.

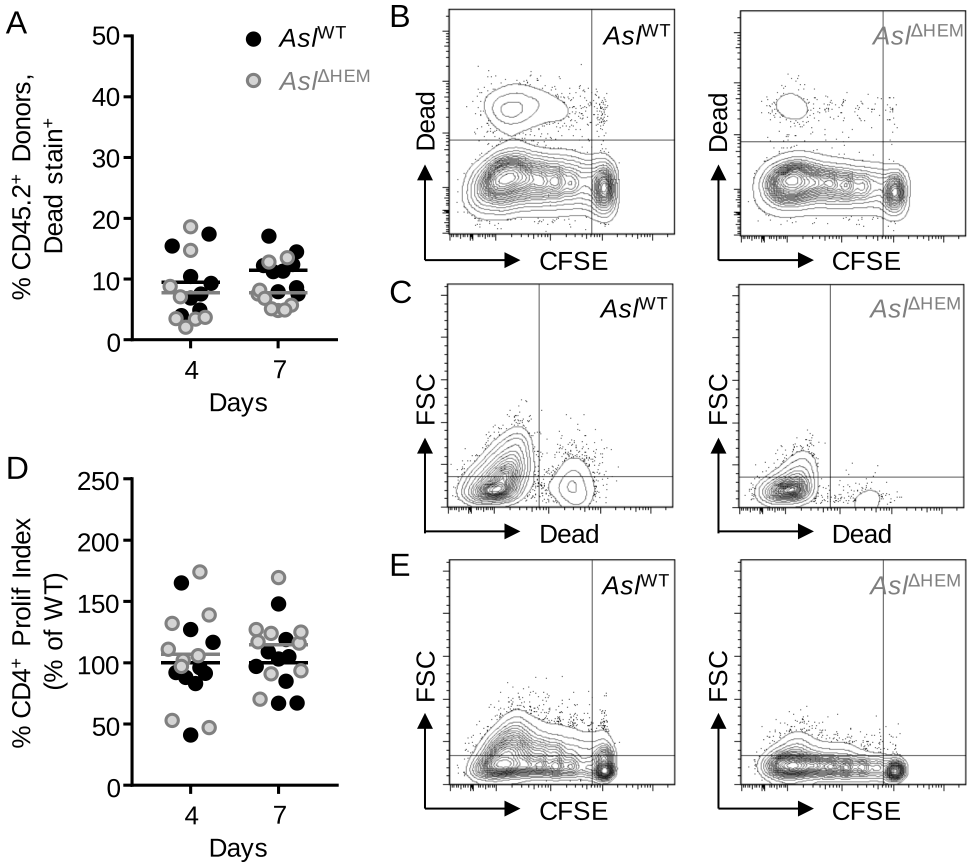

Figure S6. *Asl*^{WT} and *Asl*$^{\Delta HEM}$ P25 T cells do not differ in viability or CFSE dilution. CD4^{+} T cells were collected from P25k;*Asl*$^{flox/flox}$;Tie2-cre (*Asl*$^{\Delta HEM}$) and P25k;*Asl*$^{flox/flox}$;(-) (*Asl*^{WT}) mice and labeled with CFSE. Cells were mixed at a 50:50 ratio and transferred i.v. to C57Bl/6 mice (N ≥ 7) one day prior to *M. bovis* BCG infection (i.n., ~5×10^{6} CFUs). Transferred cells from the lung-draining mLNs were isolated at 4 and 7 days post infection by congenic markers. Dead *Asl*^{WT} (black) and *Asl*$^{\Delta HEM}$ cells (gray) indicated by the viability dye (Dead^{+}) gate are shown as frequency of CD45.2^{+} dead^{+} cells (**A**) and representative histograms (**B, C**). Proliferation data are presented as proliferation index of transferred CD4^{+} T cells (**D**) and representative histograms (**E**). Data are combined from two experiments.

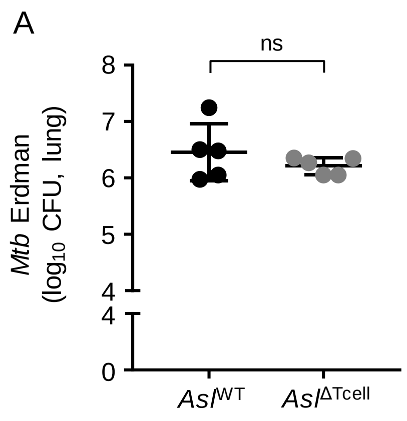

Figure S7. *Mtb* burden in *Asl*^{WT} and *Asl*$^{\Delta\mathrm{Tcell}}$ mice. (**A**) *Asl*^{WT} and *Asl*$^{\Delta\mathrm{Tcell}}$ animals were infected with ~10^2 aerosol CFU *M. tuberculosis* Erdman. Four weeks post infection, mice were sacrificed and *Mtb* colonies were quantified in the lungs. Data are from one experiment (*Asl*^{WT} N=5, *Asl*$^{\Delta\mathrm{Tcell}}$ N=5). Error bars, SD. ns = not significant by Student's t test.
